# Targeting Asparagine and Serine Metabolism in Germinal Centre-Derived B Cells Non-Hodgkin Lymphomas (B-NHL)

**DOI:** 10.3390/cells10102589

**Published:** 2021-09-29

**Authors:** Zuhal Eraslan, Grigorios Papatzikas, Jean-Baptiste Cazier, Farhat L. Khanim, Ulrich L. Günther

**Affiliations:** 1 Institute of Clinical Sciences, University of Birmingham, Birmingham B15 2TT, UK; eraslanzuhal1@gmail.com (Z.E.); f.l.khanim@bham.ac.uk (F.L.K.); 2 Institute of Cancer and Genomic Sciences, University of Birmingham, Birmingham B15 2TT, UK; gr.papatzikas@gmail.com (G.P.); J.Cazier@bham.ac.uk (J.-B.C.); 3 Centre for Computational Biology, University of Birmingham, Birmingham B15 2TT, UK; 4 Institute for Chemistry and Metabolomics, University of Lübeck, 23562 Lübeck, Germany

**Keywords:** metabolism, metabolomics, non-Hodgkin lymphomas

## Abstract

BL and DLBCL are subtypes of B-cell lymphomas that arise from germinal centre B lymphocytes. Differentiation between BL and DLBCL is critical and can be challenging, as these two types of cancer share the same morphological, immunophenotypic, and genetic characteristics. In this study, we have examined metabolism in BL and DLBCL lymphomas and found distinctive differences in serine metabolism. We show that BL cells consume significantly more extracellular asparagine than DLBCL cells. Using a tracer-based approach, we find that asparagine regulates the serine uptake and serine synthesis in BL and DLBCL cells. Calculation of Differentially Expressed Genes (DEGs) from RNAseq datasets of BL and DLBCL patients show that BL cancers express the genes involved in serine synthesis at a higher level than DLBCL. Remarkably, combined use of an inhibitor of serine biosynthesis pathway and an anticancer drug asparaginase increases the sensitivity of BL cells to extracellular asparagine deprivation without inducing a change in the sensitivity of DLBCL cells to asparaginase. In summary, our study unravels metabolic differences between BL and DLBCL with diagnostic potential which may also open new avenues for treatment.

## 1. Introduction

Burkitt lymphoma (BL) and diffuse large B-cell lymphoma (DLBCL) represent mature high-grade aggressive B-cell non-Hodgkin lymphomas (B-NHLs) originating from the germinal centre [1]. Although gene expression profiling (GEP) has revealed distinct gene expression patterns for BL and DLBCL, in practice they are difficult to diagnose intermediate forms of aggressive B-NHLs [2,3,4]. The distinction between BL and DLBCL is critical, because treatments for the two diseases differ considerably. A regimen of four drugs known as R-CHOP (rituximab, cyclophosphamide, doxorubicin, vincristine, and prednisone) is typically used as a first-line treatment for DLBCL [5], whereas a more intensive chemotherapy regimen is required for the treatment of BL [6]. Further insight into the biology of BL and DLBCL is required to reliably discern these cases and to develop novel targeted therapies that provide more effective and less toxic therapies. Here we have used metabolomics linked to transcriptomics data to show distinctive differences in asparagine metabolism between BL and DLBCL which can potentially be exploited for new treatment strategies.

Cancer cells rewire metabolic pathways to fulfil the increased nutritional demand for cell proliferation [7]. A range of haematological cancers exhibit high rates of asparagine consumption to maintain their malignant growth by supporting macromolecular biosynthesis [8,9]. Asparagine has been linked to mTORC1 activity and has been shown to regulate amino acid uptake, in particular for serine [10]. Serine represents a major source of one-carbon units, which are essential precursors for nucleotide biosynthesis [11]. Many cancer cells highly depend on serine to maintain a functional nucleotide pool to support rapid cellular proliferation. For this reason, limiting the availability of extracellular serine, or inhibiting the synthesis of serine from glycolytic intermediates, has been considered as a novel therapeutic opportunity [12]. Cancer types which lack the ability to synthesize asparagine are highly sensitive to extracellular asparagine depletion [13]. For some cancers, such as acute lymphoblastic leukemia (ALL), the reduction of extracellular asparagine through the administration of L-asparaginase (ASNase) dramatically improves clinical outcomes [13,14].

Here we compared the effect of exogenous asparagine on serine metabolism for BL vs. DLBCL. For this, we have investigated the effects of asparagine deprivation on serine uptake and production in combination with the inhibition of serine synthesis by targeting phosphoglycerate dehydrogenase (PHGDH).

## 2. Materials and Methods

### 2.1. Cell Culture 

Burkitt’s lymphoma (Glor, BL31, Sav, Ezema, Dante) and diffuse large B cell lymphoma (Farage, SUDHL4, SUDHL5, SUDHL6) were purchased from DSMZ (Braunschweig, Germany). Cell lines were maintained in exponential growth in RPMI-1640 media (Gibco-Invitrogen Ltd., Paisley, UK) supplemented with 10% fetal bovine serum (FBS, Gibco-Invitrogen), penicillin (100 U/mL) and streptomycin (100 μg/mL) (Gibco-Invitrogen) at 37 °C with 5% CO_2_. The cultures were routinely passaged every 2 days to maintain exponential phase. Cells were authenticated regularly with the NorthGene service for STR profiling. The mycoplasma test was performed with DAPI stain (Sigma Aldrich, St. Louis, MO, USA).

### 2.2. Preparation of Customised Complete RPMI-1640 Medium

Modified RPMI-1640 medium powder without glutamine, without amino acids, and without glucose was purchased from MyBioSource.com (US). Amino acid and glucose stock solutions were prepared separately in ddH_2_O to be added to the media. A nutrient mixture consisting of glucose and selected amino acids based on the composition of RPMI-1640 media formulation was prepared. For this 3.70 g of RPMI-1640 medium powder devoid of l-glutamine, amino acids and glucose were dissolved in 450 mL ddH_2_O. 1 g of NaHCO_3_ was added. Finally, the amino acid mixture, after filtration through a 0.22 µm, was added to the medium. To bring the final volume to 500 mL, ddH_2_O was added before adjusting the pH to 7.0 by addition of HCL or NaOH.

### 2.3. Isotope Labelling

For tracer-based metabolic analysis, cells at a density of 0.5 × 10^6^ cells/mL were incubated for 24 h at 37 °C with 5% CO_2_ in RPMI medium prepared with [U-^13^C] glucose (Cortecnet, Les Ulis, France), [3-^13^C] glutamine (Sigma Aldrich) and [U-^13^C]-serine (Cortecnet, Les Ulis, France) as isotope-labelled metabolites. After 24 h of incubation, cells were centrifuged at 1500 rpm for 5 min at room temperature to collect cell pellets and media samples.

### 2.4. Cell Viability Assay

Asparaginase and NCT503 were purchased from Sigma Aldrich. Asparaginase solution (100 U/mL) was prepared in distilled water. NCT503 solution (50 mM) was prepared in DMSO. Approximately 20,000 cells were plated into each well of a 96-well plate. Cells were treated with either amino acid deprived media or asparaginase at 0.1 U/mL and/or NCT503 at 10 µM. Viability was assessed using CellTiter-Blue^®^ (Promega, Fitchburg, WI, USA) with fluorescence intensity (Excitation = 560 nm, Emission = 600 nm) measured using a Victor2 plate reader (PerkinElmer, Waltham, MA, USA).

### 2.5. Western Blotting

Cells were cultured in either control media or media supplemented with asparaginase at 0.1 U/mL for 24 h before washing with cold PBS and lysing in a RIPA buffer. Fifty micrograms of protein were separated on 4–20% SDS-PAGE gradient gels. Proteins were transferred to an Immobilon-P membrane (Millipore Corp, Bedford, MA, USA) for an hour. Membranes were blocked in 5% skimmed milk in PBS-T and probed with primary antibodies overnight at 4 °C (anti-PHGDH- Sigma-Aldrich, anti-β-actin-Sigma-Aldrich) followed by secondary anti rabbit (Sigma-Aldrich) or anti mouse (Sigma-Aldrich).

### 2.6. NMR Sample Preparation

All polar extracts were dissolved in 50 μL 100 mM sodium phosphate buffer (pH 7.0) prepared with 90% H_2_O/10% D_2_O or 100% D_2_O (99.9% pure; GOSS Scientific Instruments Ltd., Crewe, UK), 3 mM sodium azide (NaN_3_), and 0.5 mM timethylsilyl-propanoic acid (TMSP, Cambridge Isotope Laboratories, Tewksbury, MA, USA) as a chemical shift reference. 35 μL of sample was transferred into 1.7 mm NMR tubes. Media samples were prepared using 162 μL of the previously saved media, and re-suspended in 18 μL of metabolomics 1M phosphate buffer to yield a final concentration of 100 mM phosphate at pH 7.0, 0.5 mM TMSP, and 3 mM NaN_3_ in D_2_O. Samples were transferred into 3.0 mm NMR tubes.

### 2.7. NMR Data Acquisition

All 1D ^1^H-NOESY and ^1^H-^13^C HSQC spectra for cell extracts were acquired at 300 K using a Bruker 600 MHz spectrometer (Bruker Biospin, Billerica, MA, USA), equipped with a 1.7-mm TCI probe and a cooled Bruker SampleJet autosampler. ^1^H 1D spectra of cell extracts were obtained using the 1D NOESY pulse sequence (noesygppr1d). Key parameters were as follows: spectral width 7183.9 Hz; 32,768 complex points, an interscan delay of 4 seconds; a mixing time of 10 milliseconds, and 128 scans. For media samples, we used a 5-mm TXO cryogenically cooled probe at 600 MHz and 32 scans.

^1^H-^13^C HSQC spectra were obtained using a modified version of the Bruker pulse program hsqcgphprsp. The spectral width for 2D ^1^H,^13^C spectra was set to 7812.5 Hz for the ^1^H observe dimension, while 24,154.6 Hz was set to for ^13^C observe dimension. For the ^1^H dimension of 2D HSQC spectra, 1024 complex data points were acquired. Spectra were acquired with 2 transients and an interscan delay of 1.5 s and non-uniform sampling (NUS) for incrementation sampling 25% of the 8192 complex points using a Hyberts schedule [15].

### 2.8. Analysis of NMR Spectra

The NMRLab/MetaboLab software [15,16] was used to process all 1D NMR data as previously described [17]. After phase correction, the icoshift software was used for segmental alignment [18]. Spectra were scaled using a probabilistic quotient normalisation (PQN-scaling). For multivariate analysis, the generalised logarithm (glog) transform was applied. Subsequently, a principal component analysis (PCA) was carried out using mean centred spectra employing PLS toolbox (Eigenvector Research). Chenomx NMR Suite (version 5.0) (Edmonton, AB, Canada, 2015) was used to assign resonances of metabolites. The intensity of metabolite signals was determined by semi-manual integration (ITN tool) in MetaboLab. Finally, metabolite intensities were normalised according to cell number.

2D HSQC spectra were processed using the NMRPipe software, version 9.2 [19] with the Hyberts extension from processing of NUS spectra [19], and were loaded into MetaboLab within Matlab, version R2017a (MathWorks, MA, USA). Cosine-squared window functions were applied to both dimensions and spectra were phased manually. Calibration was carried out manually using the L-lactic acid methyl signal as a chemical shift reference (δ 1.31/22.9 ppm). Metabolite identification was performed using the MetaboLab library [16]. PQN scaling factors from the corresponding 1D ^1^H spectra were used to scale the associated 2D HSQC spectra for both labelled and unlabelled samples.

### 2.9. Transcriptomic Data and RNA-Seq Data Analysis

Publicly available gene expression (RNA-seq) data of 19 BL [20] and 12 DLBCL [21] primary tumours were downloaded from the Sequence Read Archive (SRA) database [22]. The accession number for the BL study is SRP062178, and SRP1000105 for DLBCL. Data from primary tumours was used to avoid bias from a repeated passage in cell culture (also see Figure S2). Both studies generated paired-end RNA-seq data with the Illumina HiSeq2000 platform. The transcriptomic datasets were analysed with the Kallisto-Sleuth computational workflow [23]. For the public datasets, raw RNAseq data were downloaded in sra format from the SRA database and converted to fastq format using the SRA Toolkit 2.9.2 [24]. FastQC generated diagnostic plots which were examined for per base sequence distribution, GC%, per sequence quality distribution, and vector or adapter contamination. RNAseq data with both R1 and R2 read-pair were selected for further analysis based on: the per base sequence quality score with median for any base was ≥25; the averaged quality score per read was ≥20 (this equates to a 1% error rate); and the absence of adapter contamination. Reads were aligned to the GRCh38 human reference genome cDNA index (Ensembl rel.99) and counted to quantify for 39,320 transcript abundances corresponding to 17,048 genes with the Kallisto 0.43.0 software [25]. Gene-level differential expression analysis was performed with the Sleuth 0.30.0 R statistical package, comparing BL to DLBCL cases. Differentially expressed genes (DEGs) were calculated with the Wald statistical test, correcting for multiple comparisons with the Benjamini-Hochberg method using a false discovery rate (FDR) threshold of 1% (q values < 0.01). Ensembl gene transcripts were annotated to Entrez IDs, official gene symbols and KEGG enzymes with the BioMart 2.40.3 R statistical package [26]. Transcripts per million (TPM) expression values were calculated to normalise for sequencing depth and gene length. Log2TPM+1 values were used in the unsupervised method Principal Component Analysis (PCA) with the PCAtools 1.0.0 R statistical package to identify clusters and outliers within the data. Furthermore, a list of 2.552 metabolic enzymes was retrieved from the global KEGG metabolic network for human [27] to study metabolic genes in PCA. The log2TPM + 1 expression values were also used in heatmaps and in hierarchical clustering with the Ward method and a distance of 1–Spearman’s rank correlation.

## 3. Results

### 3.1. BL Cells Consumed Dramatically More Extracellular Asparagine Than DLBCL and Regulates Serine Metabolism

**Figure 1:**
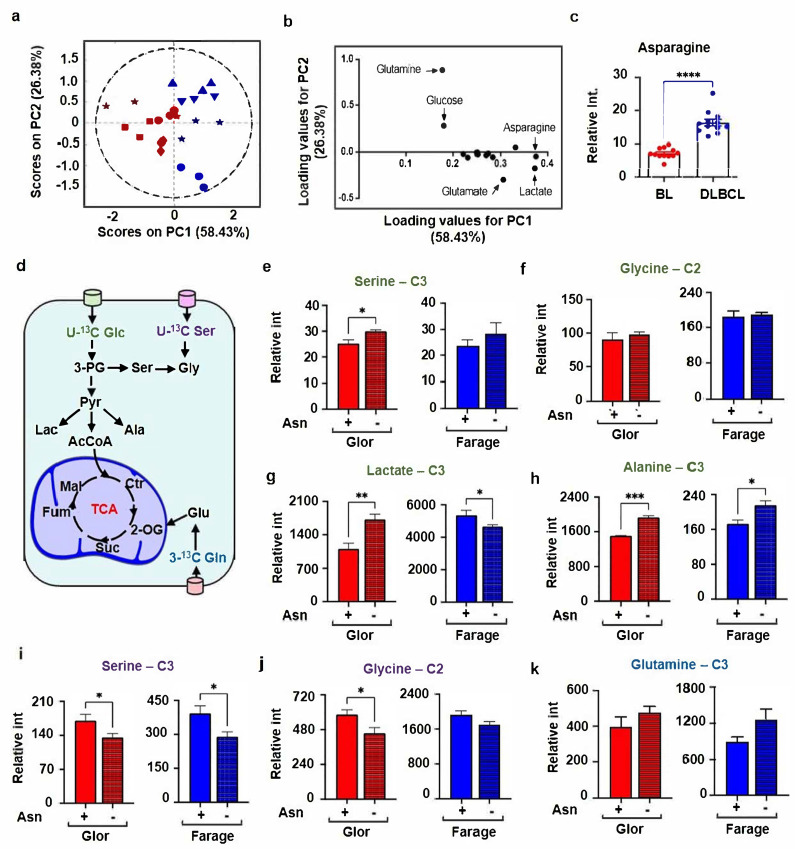
Regulation of intracellular metabolism by exogenous asparagine. (**a**) Principal Component Analysis (PCA) for 1D ^1^H-NMR for media samples using a metabolite data matrix consisting of 13 metabolites in the spent media of Sav, Glor, Ezema, BL31 representing BL (red) and Farage, SUDHL4, SUDHL5 and SUDHL6 cells representing DLBCL (blue). (**b**) Representation of corresponding loadings plot. (**c**) Comparison of 1D ^1^H-NMR peak intensities of asparagine at 2.80 ppm for the media samples of DLBCL and BL cells line cells. (**d**) Simplified illustration of metabolism of the labelled precursors [U-^13^C] glucose, [U-^13^C] serine and [3-^13^C] glutamine (**e**–**k**). ^13^C peak intensity of the metabolites derived from either [U-^13^C] glucose labelled media (**e**–**h**) or [U-^13^C] serine and [3-^13^C] glutamine labelled media (**i**–**k**) in the absence or presence of asparagine for 24 h. The confidence ellipse in the PCR score plot is based on 95% confidence. Bar graphs represent mean ± SEM, with n = 3. Data are shown as * *p* < 0.05; ** *p* < 0.01; *** *p* < 0.001, **** *p* < 0.0001.

To elucidate metabolic differences between BL and DLBCL, metabolites in cell culture were studied in the media of BL and DLBCL cell using NMR spectroscopy. Using 1D ^1^H-NMR spectroscopy, we assigned 13 key signals in media samples for a subsequent principal component analysis (PCA). Media samples were obtained for Sav, Glor, Ezema, BL31 representing BL, and for Farage, SUDHL4, SUDHL5 and SUDHL6 cells, representing DLBCL. The PCA score plot of the 1D ^1^H-NMR spectra is shown in Figure 1a. The first principal component (PC1) describes 58% of the total variation, while PC2 demonstrates 26% of the total variation. The PCA pronouncedly separated BL cells from the DLBCL cells on PC1, reflecting the differences in extracellular metabolite composition. The loadings plot of the PCA analysis showed that that the separation of BL from DLBCL was mainly based on differences in relative amounts of asparagine (Figure 1b). Univariate analysis confirmed that BL cells consumed dramatically more extracellular asparagine and glutamine than the DLBCL cells (Figure 1c and Figure S1). 

Considering that a recent report claims that serine metabolism is regulated by asparagine in a liposarcoma cell line, LPS2 [10], we aimed to further investigate the role of asparagine on metabolism in BL and DLBCL cells. To determine the influence of extracellular asparagine on glycolysis, serine and glutamine uptake, BL and DLBCL cells were cultured in a medium supplemented with U-^13^C-stable-isotope labelled glucose or with ^13^C-labelled serine and 3-^13^C-labelled glutamine and subsequently analysed using 2D HSQC NMR experiments. Glor and Farage cell lines were selected as representatives of BL and DLBCL, respectively. Firstly, to assess the effect of asparagine on glycolysis, BL and DLBCL cells were cultured for 24 h in asparagine depleted vs. complete media, both containing [U-^13^C]-glucose. For this, we used HSQC spectra and cells with different stable-isotope labelled metabolic precursors. Intensities in HSQC spectra were compared between samples with (+) and without (−) asparagine in the media. HSQC analysis of cells grown media with [U-^13^C]-glucose revealed that asparagine deprivation induced an approximately 20% increase in the formation of serine from [U-^13^C]-glucose in both Glor and Farage cells (Figure 1e). However, glycine production from [U-^13^C]-glucose remained steady in both cell lines (Figure 1f). Compared to cells grown in culture media with asparagine, a substantial increase in production of lactate from glucose was observed in Glor cells, while a decrease was found for lactate production in Farage cells (Figure 1g). Moreover, asparagine deprivation also resulted in an enhancement in alanine production in Glor and Farage cells (Figure 1h).

To determine the influence of asparagine on serine and glutamine uptake from media, BL and DLBCL cells were cultured in asparagine depleted or complete media containing [U-^13^C]-serine and [3-^13^C]-glutamine for 24 h. An HSQC analysis of Glor and Farage cells revealed that the influx of serine was reduced by 25% in both Glor and Farage cells in asparagine depleted media (Figure 1i). We also observed a marked change in the level of glycine originating from extracellular [U-^13^C]-serine (Figure 1j). The use of [3-^13^C]-glutamine to test whether asparagine regulates glutamine uptake revealed that absence of asparagine slightly raised the intracellular level of ^13^C-glutamine levels (Figure 1k). These results suggest and confirm that asparagine regulates glucose metabolism, serine metabolism and glutamine uptake in BL and DLBCL cells [10].

### 3.2. BL Tumours Differentially Express the Genes Involved in Serine Metabolism Compared to DLBCL

**Figure 2:**
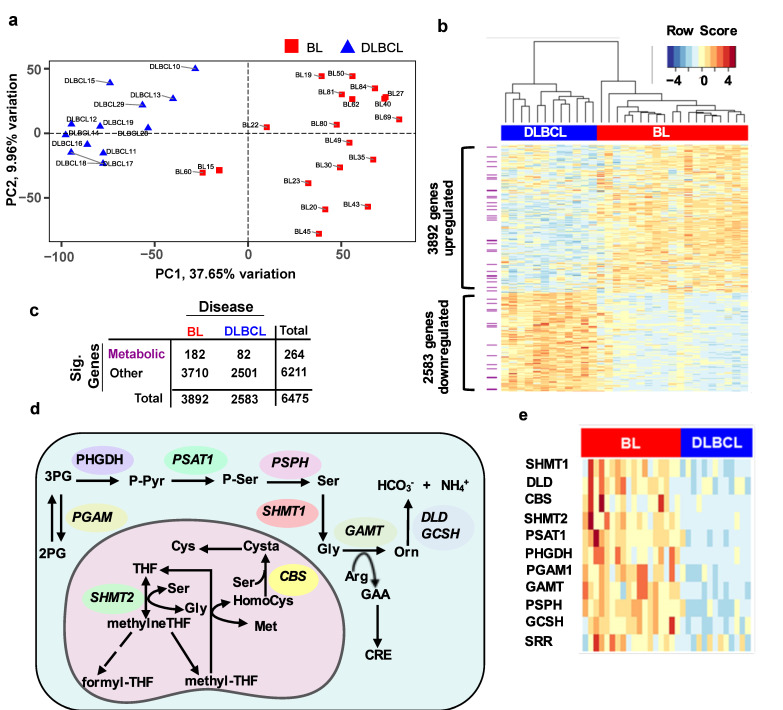
Genetic differences between BL and DLBCL. Principal component analysis performed on transcriptome profiles of 19 endemic BL and 12 GCB-DLBCL cases. (**a**) The first and second principal components are contributing to 37.65% and 9.96% of the total explained variation, respectively. The marker shapes represent the origin of isolated malignant B-cells: abdomen (circle), bone marrow (BM, triangle), jaw (square), lymph nodes (LN, cross), neck (square cross) and pelvic (star). (**b**) The 6475 statistically significant altered genes (FDR < 0.01) from differential expression analysis are visualised in a heatmap. Gene expression values have been converted to a Z-score scale along the rows for case comparisons. The dendrogram in hierarchical clustering analysis was produced with the Ward method and distance 1-Spearman’s rank correlation. (**c**) Contingency table used for two-sided Fisher’s Exact test to compare the relative proportions of significant genes between the BL and DLBCL. (**d**) Illustration of the genes involved in serine metabolism. (**e**) Heatmap of statistically significant genes associated with serine metabolism in DLBCL and BL. Abbreviations: SHMT1: serine hydroxy methyltransferase 1; DLD: dihydrolipoamide dehydrogenase; SHMT2: serine hydroxy methyltransferase2; CBS: cystathionine beta synthase; PSAT1: Phosphoserine aminotransferase; PHGDH: phosphoglycerate dehydrogenase; PGAM1: phosphoglycerate mutase 1; GAMT: guanidinoacetate methyltransferase; PSPH: phosphoserine phosphatase; GCSH: glycine cleavage system protein H; SRR: serine racemase. Dendrogram in hierarchical clustering analysis was produced with Ward method and distance 1- Spearman’s rank correlation.

To explore whether these metabolic observations are linked to genetic features, we looked at differences in the gene expression profiles between BL vs DLBCL primary tumours. For this, we analysed the transcriptome profiles of 19 endemic BL and 12 germinal centre B-cell like (GCB)-DLBCL cases using publicly available RNA sequencing data from the SRA database. Dimensionality reduction with PCA was performed using expression values from 17,048 genes to explore transcriptomic associations between the two diseases. PC1, which explains 37.65% of the variation, is the optimal component to segregate BL from DLBCL (*t*-test for PC1: *p* = 4.96 × 10^−13^, q = 5 × 10^−13^), suggesting that the two diseases are transcriptionally distinct. (Figure 2a). Differential expression analysis identified 6,475 genes, which were expressed differently between the two diseases (Figure 2b). A contingency table was created to test if the relative proportions of metabolic and other genes were the same in the two diseases. A two-sided Fisher’s exact test was applied and revealed that there was a significant difference (*p* value = 0.0025, odds ratio = 0.66) between BL and DLBCL in metabolic and other significant genes (Figure 2c). Notably, 264 statistically significant metabolic genes were found (Figure 2c). To test whether metabolic genes are expressed in BL vs. DLBCL, we constructed gene interaction networks, using the STRING database [28] (Figure 2d). We found that all the genes involved in serine metabolism were significantly upregulated in BL cases in comparison with DLBCL (Figure 2e).

### 3.3. Asparaginase and NCT503 Reduce Extracellular Asparagine and Serine Production from Glucose Respectively

**Figure 3:**
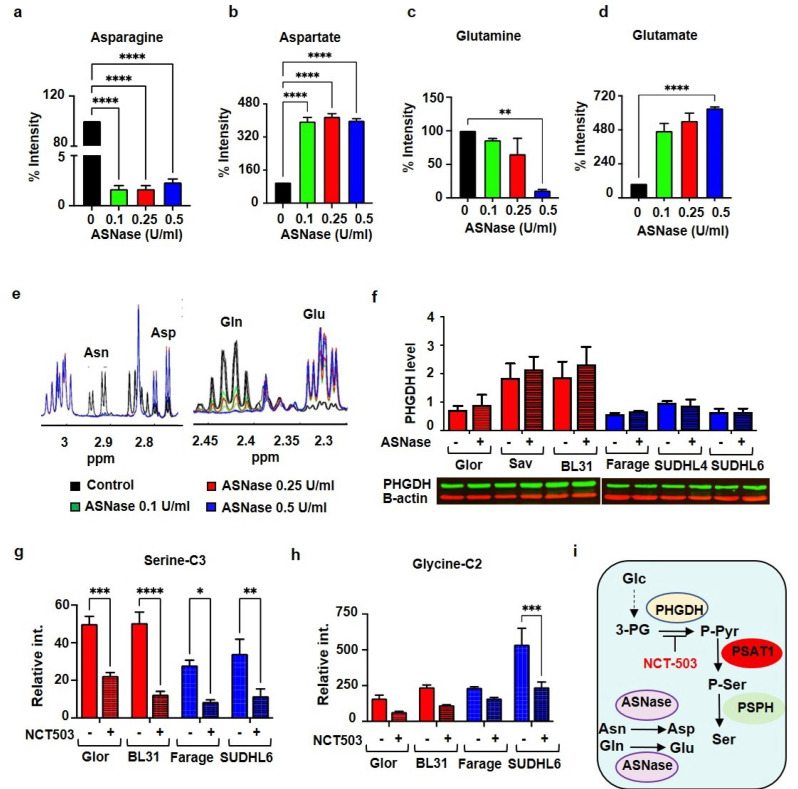
Comparison of serine metabolism in BL and DLBCL. Relative 1D ^1^H-NMR peak intensity of (**a**) asparagine, (**b**) aspartate, (**c**) glutamine and (**d**) glutamate in the fresh medium supplemented without or with asparaginase (ASNase) at 0.1 U/mL, 0.25 U/mL and 0.5 U/mL for 24 h. (**e**) Representative 1D NMR spectra demonstrating the alteration in the level of asparagine, aspartate, glutamine and glutamate in medium supplemented with no ASNase, ASNase at 0.1 U/mL (green line), 0.25 U/mL (red line), 0.5 U/mL (blue line) for 24 h. (**f**) Western blot analysis showing the expression of PHGDH in Glor, Sav, BL31, Farage, SUDHL4 and SUDHL6 cell lines after treatment without or with 0.1 U/mL ASNase for 24 h. PHGDH level was normalized to β-actin. Analysis of 2D ^1^H-^13^C HSQC spectra of (**g**) serine and (**h**) glycine extracted from Glor, BL31, Farage and SUDHL6 treated with NCT-503 at 10 μM. Bar graphs represent mean ± SEM, with n = 3; * *p* < 0.05, ** *p* < 0.01; *** *p* < 0.001, **** *p* < 0.0001. (**i**) Illustration of the effects of ASNase and NCT503.

The use of asparagine-free medium is not sufficient to eliminate the availability of asparagine as cells can generate asparagine by other means, e.g., from aspartate by transamination. Therefore, asparaginase (ASNase), which catalyzes the deamination of asparagine to aspartate, has been used to decrease extracellular asparagine in asparagine dependent cancers. Thus, to test whether and to what extent ASNase can completely diminish the extracellular asparagine, ASNase at different doses was added to fresh complete media. After the supplementation of fresh media with ASNase at 0.1 U/mL, 0.25 U/mL, and 0.5 U/mL for 24 h, 1D ^1^H-NMR measurements were acquired and asparagine levels were determined. ASNase at 0.1 U/mL was found to almost completely convert asparagine into aspartate in comparison with the control media (Figure 3a,b). Moreover, albeit to a lesser degree, ASNase also converted glutamine into glutamate in a dose-dependent manner (0.1 U/mL caused a reduction by 14%, 0.5 U/mL by 88% within 24 h) (Figure 3c,d). Thus, ASNase at 0.1 U/mL, which converted 98 % asparagine into aspartate and only 14% glutamine to glutamate, was used for subsequent experiments (see NMR spectra in Figure 3e).

We further asked whether the expression level of PHGDH, an enzyme that catalyses the first rate-limiting step of serine synthesis from glucose, is regulated by extracellular asparagine. To answer this question, BL (Glor, Sav, BL31) cells and DLBCL (Farage, SUDHL4, SUDHL6) cells were exposed to 0.1 U/mL ASNase for 24 h. Western blotting was used to evaluate the relative concentration of PHGDH in treated and non-treated cells, and showed that ASNase treatment increased PHGDH protein levels in BL cells while no change was observed in DLBCL cells (Figure 3f). Considering our observation that asparagine depletion increased serine production from glucose and the expression level of PHGDH in BL cells, we asked whether inhibition of PHGDH would decrease serine production from glucose. To answer this question, we cultured DLBCL (Farage, SUDHL6) and BL (Glor, BL31) cells with [U-^13^C]-glucose with 10 μM NCT503 as a PHGDH inhibitor for 24 h. BL and DLBCL cells exhibited a marked attenuation in the amounts of [U-^13^C]-glucose derived [3-^13^C]-serine and [2-^13^C]-glycine compared to untreated cells (Figure 3g,h). Our results indicate that while ASNase could be used to completely eliminate extracellular asparagine and reduce the concentration of glutamine, the increase in serine synthesis caused by asparagine deprivation could be reversed by the use of NCT503, a potent inhibitor of PHGDH (Figure 3i).

### 3.4. Combination of ASNase with NCT Increases the Sensitivity of BL Cells to ASNase

**Figure 4:**
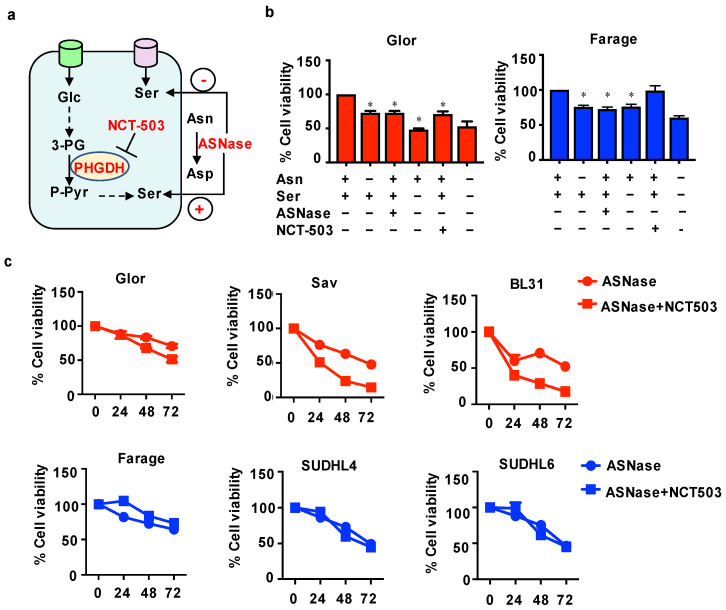
Combined effect of asparaginase and NCT503 on cell viability. (**a**) Illustration of combined effects of asparaginase (ASNase) and NCT503 on serine and asparagine metabolism. The viability of Glor and Farage cells in a complete medium, in asparagine-free medium, in serine free medium, in the medium with ASNase at 0.1 U/mL, in the medium with NCT503 at 10 μM and in the serine and asparagine free medium after 72 h (**b**). Treatment of Glor, Sav, BL31, Farage, SUDHL4, SUDHL6 with ASNase at 0.1 U/mL and ASNase at 0.1 U/mL plus NCT-503 at 10 µM for 24, 48 and 72 h (**c**). Cell viability was assessed by CellTiter Blue assay. Data is shown as mean ± SEM. * *p* < 0.05.

Considering that ASN deprivation had a marked effect on serine metabolism, we decided to explore a combined approach of conversion of exogenous asparagine by ASNase and inhibition of serine synthesis by a PHGDH inhibitor, NCT503 (Figure 4a).

Firstly, to gain insight into the effect of asparagine and serine on cell viability, BL Glor cells and DLBCL Farage cells were cultured in the absence of extracellular asparagine or serine, or in media supplemented with ASNase at 0.1 U/mL or NCT503 at 10 µM for 72 h. Asparagine deprivation and treatment of the media with ASNase at 0.1 U/mL induced a similar reduction in the viability of Glor and Farage cells (Figure 4b). Glor cells appeared to be more sensitive to serine starvation than inhibition of PHGDH enzyme, as the deprivation of serine resulted in a ~50% decrease in cell viability (Figure 4b). Nevertheless, the NCT503 treatment of Glor cells caused a ~25% reduction in cell viability in comparison with the control group. Moreover, Farage cells were found not to be affected by the inhibition of PHGDH enzyme by NCT503 compared to cells cultured in complete media. Moreover, BL cells were found to be sensitive to inhibition of serine uptake and serine production from glucose opposite to Farage cells (Figure 4b). These results suggested that BL cells are much more sensitive to the maintenance of the serine pool than DLBCL cells.

Previously, asparagine starvation was shown to decrease serine uptake and to increase serine production from glucose. BL cells were found to be more dependent on extracellular asparagine and to also produce more serine from glucose compared to DLBCL cells. In the light of these findings, we aimed to test if the combination of NCT503 with ASNase could increase the sensitivity of BL cells to ASNase. To answer this question, BL (Glor, Sav, BL31) and DLBCL (Farage, SUDHL4, SUDHL6) cells were treated with ASNase at 0.1 U/mL or combination of ASNase at 0.1 U/mL with NCT503 at 10 µM for 24, 48 and 72 h. ASNase alone reduced the viability of BL and DLBCL cells to around 50% after 72 h of treatment (Figure 4c). However, the combination of ASNase with NCT503 had a synergistic effect on cell viability only in BL cells, exhibiting further decrease in cell viability as compared to those obtained from treatment of ASNase alone. Conversely, the combination of ASNase with NCT503 showed no synergistic effect on the viability of DLBCL cells, suggesting that the combination of ASNase with NCT503 is solely synergistic for BL.

## 4. Discussion

Recent research has started to unveil novel traits in metabolic pathways in haematological cancers [9], although little is known about metabolism in germinal centre derived B lymphomas. Dependence of tumours on selected metabolic precursors has led to the current paradigm, which is that asparagine coordinates serine metabolism and nucleotide synthesis [10]. In our study, we confirm that extracellular asparagine is critical for serine uptake and the synthesis of serine from glucose, as well as defining a new treatment model which solely works for BL. Serine is an important amino acid, mainly for its contribution to one carbon metabolism, providing carbon units for nucleotide synthesis [29].

We show that BL cells are considerably more dependent on extracellular asparagine than DLBCL cells and also express the genes involved in serine metabolism at a higher level than DLBCL cells. While differences in metabolic genes have previously been shown between BL and DLBSCL cells [30,31], the specific role of serine has not been described.

Although asparagine has been shown to regulate serine uptake, we also found that asparagine starvation caused an increase in serine production from glucose, most likely as a compensatory mechanism, thus highlighting the essential role of serine. Many studies have demonstrated that several types of haematological cancers are addicted to exogenous asparagine owing to reduced or complete loss of expression of asparagine synthetase (ASNS). In the case of ALL, ASNase is standardly used as part of treatment [13]. Despite the use of ASNase for the treatment of ALL patients, multi-agent chemotherapy regimens used in BL do not include ASNase. Here, we show that ASNase at very low dose could be used to treat BL, especially in combination with a PHGDH inhibitor. This is also confirmed by genetic analysis, which shows that genes involved in serine biosynthesis are expressed at higher levels in BL compared to DLBCL. Therefore, PHGDH must be considered as an attractive new target for BL treatment.

## 5. Conclusions

Given the fact that BL is characterised by chromosomal rearrangements of the c-Myc proto-oncogene, which activates the expression of several enzymes in serine biosynthesis, the upregulation of genes involved in serine metabolism in BL patients compared to DLBCL patients might be resulted from the overexpression of c-Myc. These findings highlight the metabolic differences between BL and DLBCL, and the dependence of BL cells on extracellular asparagine and serine. Further investigations of whether simultaneous targeting of asparagine uptake along with inhibition of the serine biosynthesis pathway might lead to an effective new treatment avenue for BL are warranted. Further work is also needed to show whether a similar dependence on serine is found in the clinically relevant DLBCL NOS, which constitutes almost one third of all NHL.

## Data Availability

Metabolomics data can be obtained from the authors.

## Supplementary Materials

The following are available online at https://www.mdpi.com/article/10.3390/cells10102589/s1, Figure S1: Selection of cell lines; Figure S2: Effect of long-term culturing of cells.
