# Supplementary material for: Targeting Asparagine and Serine Metabolism in Germinal Centre-Derived B Cells Non-Hodgkin Lymphomas (B-NHL)
Source: Cells. 2021 Sep 29;10(10):2589. doi: 10.3390/cells10102589 (PMC8533740; doi:10.3390/cells10102589)
Supplement: Supplementary file 1 [file cells-10-02589-s001.zip › cells-1340465-supplementary.pdf]

Selection of cell lines

Cell lines were selected because they were representative for the responses that we saw with each tumor type. Experiments with Farage and Glor identified the importance of asparagine and serine. Particularly, Farage and Glor cells were found to be least asparagine dependent DLBCL and BL cells respectively (see Figure 1 below). We would be happy to make this Figure available as Supplementary

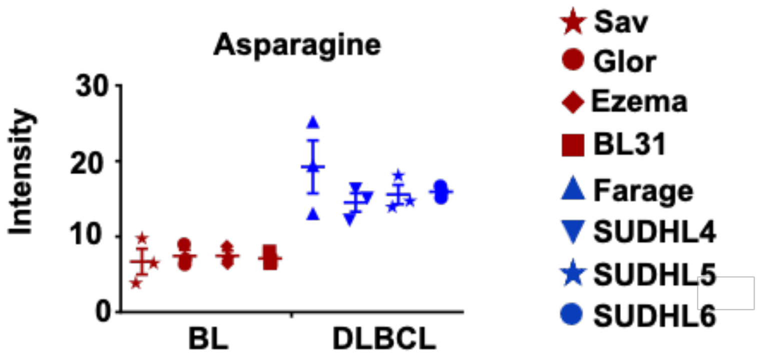

Figure S1. Selection of cell lines. Representation of asparagine levels in the media of BL and DLBCL. Comparison of 1D ^{1}H-NMR peak intensities of asparagine at 2.80 ppm for the media samples of Sav, Glor, Ezema, BL31 cells representing BL; and Farage, SUDHL4, SUDHL5 [illegible]

Effect of long-term culturing of cells

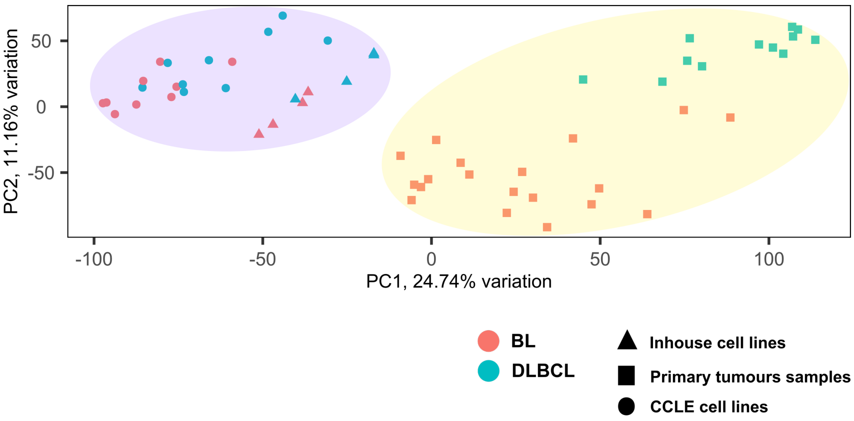

Figure S2. Effect of long-term culturing of cells. Unsupervised principal component analysis with transcriptomic data from BL/DLBCL cell lines and primary tumours. The first and second principal components are contributing to 24.74% and 11.16% of the total explained variation, respectively. The marker shapes represent inhouse cell lines (triangle) and primary tumours (square) from chapter 3 and CCLE cell lines (circle) from chapter 4. The purple circle includes only cell lines samples, while the yellow circle only primary tumours samples
